# Engagement in physical activity, suicidal thoughts and suicide attempts among older people in five developing countries

**DOI:** 10.7717/peerj.7108

**Published:** 2019-06-12

**Authors:** Bishwajit Ghose, Ruoxi Wang, Shangfeng Tang, Sanni Yaya

**Affiliations:** 1 School of International Development and Global Studies, University of Ottawa, Ottawa, Canada; 2 School of Medicine and Health Management, Tongji Medical College, Wuhan, Hubei, China

**Keywords:** Suicidal thoughts, Suicidal attempts, Physical activity, Elderly individual

## Abstract

**Background:**

Suicide causes rising economic costs and public health risks for communities in the worldwide. Physical activity (PA) is considered a potentially feasible approach to reduce risk of suicide with low cost and high accessibility, and therefore attracting increasing attention. However, current literature on the association between PA and suicidal behavior amongst elderly people in low- and middle-income countries (LMICs) are scarce. Therefore, in this study we aimed to examine the relationship between suicidal thoughts (ST) and suicidal attempts (SA) with PA among elderly people in five LMICs.

**Methods:**

Cross-sectional data were collected from WHO’s Study of Global Ageing and Adult Health (SAGE) with 2,861 participants aged 50 years or above. Variables included: self-reported occurrence of ST and SA during past 12 months and four types of PA (vigorous physical activity (VPA), moderate physical activity (MPA), walking/bike riding, moderate leisure time physical activity (MLPA)).

**Results:**

The overall prevalence of taking >75 min of VPA/week, >150 min/week, MLPA and walking/bike riding were, respectively, 85.4% (95% CI [81.3–88.7]), 61.6% (95% CI [52.9–69.6]), 9.6% (95% CI [7.2–12.6]) and 75.1% (95% CI [68.7–80.6]). Respectively, 31.0% (95% CI [24.3–38.7]) and 5.5% (95% CI [3.9–7.5]) of the respondents reported having morbid thoughts and SA during last 12 months. In adjusted multivariable regression analysis, not engaging in PA revealed positive association with higher odds of having morbid thoughts and SA, however, with varying degrees for different types of PA among men and women and across countries. The adjusted odds ratio among elderly who encountered ST increased significantly with PA levels (1.265 in male and 1.509 in female with VPA, 1.292 in male and 1.449 in female with MPA, 1.669 in female with LMPA and 3.039 in women with walk/bike); similarly, with SA (1.526, 1.532, 1.474 and 1.392 in women with VPA, MPA, LMPA and Walk/bike, respectively). The degree of adjusted odds ratio varied between genders and among countries.

**Conclusion:**

Although the data were cross-sectional, and no linear dose-response relationship was observed between PA and morbid thought and suicide ideation, the findings provide important indications of potential harmful effects of no/inadequate PA on psychological morbidities among older individuals. Promoting adequate PA among older individuals through community-based suicide prevention programs can potentially contribute to reduction in the burden of PA in LMICs.

## Background

Suicide is not only a personal tragedy that causes permenent loss of life, but also a catastrophe for the families, friends and even communities left behind (World Health Organization, 2014). Suicide accounts for 1.4% of all death worldwide, ranking as the 17th leading cause of death in 2015 (World Health Organization, 2018). Besides the heavy burden that a completed suicide brings about, it is closely associated with two other non-fatal suicidal behaviors: suicidal thoughts (ST) and suicide attempts (SA), referring to thinking about death and attempting to end one’s own life, respectively. The prevalence of ST and SA is significantly higher than in completed suicide (Turecki & Brent, 2016), accounting for 9.2% and 2.7%, respectively (Nock et al., 2008). Moreover, amongst those who have had ST within the previous 12 month, nearly 15% may put the idea into practice of the people who attempted to end their lives but failed, 16.3% will repeat SA within 12 months, and 3.9% may complete suicide within 5 years (Carroll, Metcalfe & Gunnell, 2014). In other words, suicidal behaviors are strong predictors of complete suicide (Runeson et al., 2010), and therefore, important in guiding explorations on suicide prevention.

Suicide is preventable. The past few decades have witnessed a significant number of studies exploring the associated risk factors. The fruitful findings reveal that: (1) the suicide action amongst older adults involves a large variety of determinants, such as sociodemographic characteristics (including religion, gender, residency, employment status etc.) (Hawton & Heeringen, 2009), poor mental health status (including affective disorders such as depression and anxiety, etc.) (Brown et al., 2000; Waern, Rubenowitz & Wilhelmson, 2003), serious physical illness (including cancer, chronic pain, perceived health status) (Ahmedani et al., 2017) and low social functions (including hopeless, loneliness, etc.) (Deuter et al., 2016); and (2) amongst these factors, depression is a major factor (Isacsson et al., 2010).

Efforts have been made to seek effective interventions to reduce risks of ST and (or) SA. Prior explorations were mainly led by medical understanding and frameworks, and therefore largely concentrated on medical identification and treatment of depression (Deuter et al., 2016). Interventions, such as cognitive behavioral therapy and some antidepressants, have indeed been recognized as helpful in reducing risks of ST/SA; nevertheless, many require relatively intensive resources and are confined to a limited scale of population with poor viability in many contexts (Milner et al., 2015). In response to this situation, some researchers highlighted the need to extend the medical/clinical based intervention-seeking paradigm by paying more attention to socio-environmental conditions (Deuter et al., 2016). Since then, some studies started to explore approaches that can be carried out on a larger scale, with lower costs and based on resources that already exist in the community. Based on the emerging evidence regarding its potential to reduce depressed symptoms (Du et al., 2015; Ku et al., 2012) and its accessibility, physical activity (PA) has become one of the prominent options that may help reduce the risk of suicide and promote psychosocial health especially among the elderly populations.

Among the limited number of studies that attempted to validate the hypothesis, findings turned out to be largely heterogeneous. Regarding those aimed to validate the relationship between PA and ST/SA, some have proved that engaging in PA leads to a significant decrease in risk of suicide (Davidson et al., 2013; Sturm et al., 2012), whereas some failed to do so (Arat & Wong, 2017; Bishwajit et al., 2017). Moreover, some attempted to further investigate the dose-response relationship by categorizing PA into light physical activity (LPA), moderate physical activity (MPA) and vigorous physical activity (VPA) according to WHO guidelines (Song & Lee, 2016; Wang et al., 2016) or by frequency (Elliot et al., 2012; Unger, 1997). Some studies indicated that all levels of PA are protective to suicidal behaviors (Cho, 2014; Elliot et al., 2012); nevertheless, others resulted in no significant correlation or even revealed contradictive relationships with suicidal behaviors between different levels of PA. For instance, Bailey & McLaren’s (2005) study did not find a significant relationship between PA and depression nor between PA and ST in older adults; whereas in Song & Lee’s (2016) study, activity above moderate intensity was found to be associated with higher risk of SA in female adults, and regular exercise was found to be associated with lower risk of ST in male adults. The above inconsistent findings, in turn, call for further evidence regarding this issue.

Furthermore, much of the available evidence came from developed countries and was extracted from adolescents, leaving those who lived in low- and middle-income countries (LMICs) and in their 50s or older not receiving enough attention they deserve. For instance, Vancampfort et al.’s (2018) systematic review regarding the relationship between PA and ST (published in 2018) extracted 11 studies conducted in adolescents, 15 in adults whereas only three in older adults; and these three studies were all conducted in developed countries. Statistics remind that, on the one hand, suicides in LMICs countries account for 84% of worldwide suicides, and India and China together account for nearly 50% (Phillips & Hui, 2012); on the other hand, suicide rate generally increases with age (World Health Organization, 2014). Taking into consideration the large gap between LMICs and high-income countries regarding the political and sociocultural contexts, the complexity of suicidal behaviors with unique characteristics and risk factors in later life (Conwell, Duberstein & Caine, 2002), and the growing proportion of elderly in the general population in LMICs (Shah, 2011), whether PA has an impact on ST/SA among the elderly population in LMICs awaits further investigation.

To the best of our knowledge, no study to date has explored this topic using a cross-national sample of the LMICs. Therefore, we carried out the study with the aims to explore the patterns of suicidal behaviors (ST and SA) and PA across various socio-demographic groups and to examine the relationship between different levels of PA with ST and SA in elderly adults. Since prior research indicated potential gender discrepancy on the relationship between PA and suicidal behaviors (Brown & Blanton, 2002), we have also investigated the patterns in these two subgroups. As data on suicidal behavior are rare for LMICs, we used secondary surveys from World Health Organization conducted in the following countries: China, Ghana, India, Russia and South Africa.

## Methods

### Survey details

For this study we used data from the Study of Global Ageing and Adult Health (SAGE) of WHO Wave 1 (2007–2010). SAGE is a longitudinal survey conducted in six countries including China, Ghana, India, Mexico, the Russian Federation and South Africa with an aim to provide quality data on various aspects of elderly health for population aged 50 and higher. For the present analysis we included men and women aged 50 years and above who provided information on the indicators of suicide and PA. The sample population were chosen randomly from community-dwelling households and individuals by using stratified sampling techniques. In this study, we considered all countries except Mexico due to lack of available explanatory variables. Response rates were China (95%), Ghana (92%), India (92%), Russian (71.8%), South Africa (60%). Description of study and sample design were published elsewhere (Thapa, Martinez & Clausen, 2014).

### Variables

*Outcome variables*: were self-reported occurrence ST and SA in the last 12 months period. Participants were asked: *During the last 12 months*, (1) *Have you thought of death, or wish you were dead?* And, (2) *During this period, have you ever tried to end your life?* Both of the questions had three possible answers: Yes/No/Don’t know. Participants who responded as “Yes” were considered as having experienced ST and SA, and “No” if responded otherwise.

*Main Explanatory variables*: (1) VPA, (2) MPA, (3) Leisure time moderate physical activity (LMPA), (4) Walking/bike-riding. These were measured based on the responses to the following questions:

1. VPA: *Does your work involve vigorous-intensity activity that causes large increase in breathing or heart rate (e.g., heavy lifting or chopping wood) for at least 10 min continuously?*
2. MPA: *Does your work involve moderate-intensity activity that causes small increase in breathing or heart rate (e.g., carrying light loads or cleaning) for at least 10 min continuously?*
3. LMPA: *Do you do any moderate intensity sports, fitness or recreational activities that cause large increases in breathing or heart rate (e.g., running or football) for at least 10 min continuously?*
4. Walking/bike-riding: *Do you walk or use a bicycle for at least 10 min continuously to get to and from places?*

These variables were selected based on their demonstrated associated with individual adverse psychosocial outcomes that are likely to influence ST/SA according to the international guidelines set by World Health Organization (2010). As durations of LMPA and walking/bike-riding were not available for the majority of the participants, these were categorized as “Yes” or “No” based on whether or not the individual took this form of activities.

*Control variables:* Age in years (Current age of the respondents): 50–59/60–69/70–79/79+; Residency (Place of residence of the household): Urban/Rural; Sex: Male/Female; Currently married: No/Yes; Educational attainment: Nil or less than primary school/primary/secondary high school and higher; Employed: Yes/No; Ever used alcohol: Yes/No; Income sufficiency (Perceived income status of the respondent): Not at all/Mostly/completely; **Self-rated health (Respondent’s estimation of general health status) Good/Neutral/Poor; **Satisfaction with life (Respondent’s estimation of satisfaction with life): Satisfied/Neutral/Very dissatisfied.

**Besides the sociodemographic variables, we also added self-rated health and satisfaction with life as proxy indicators of overall well-being which is likely to be associated with psychosocial health and self-harm behavior.

### Data analysis

Data were analyzed with SPSS V24. Participants with reported self-reported cognitive limitations were excluded from the analysis (<1%). Sample profile was described as percentages. Prevalence rates of the explanatory and outcome variables were presented as percentages with 95% CIs. The association between ST and SA was analyzed with binary logistic regression methods by adjusting for the potentially confounding variables. Owing to known difference in suicidal behavior between men and women, results from regression analyses (Odds Ratios) were stratified by sex and calculated separately for each of the five countries. Association between levels of PA and outcome variables was considered statistically significant for *p* < 0.05.

### Ethical approval

Study of global ageing and adult health surveys were approved by the World Health Organization’s Ethical Review Committee. Additionally, partner organizations in each country implementing SAGE obtained ethical clearance through their respective institutional review bodies.

## Results

### Sample characteristics

Sociodemographic and health indicators of the sample population were summarized in Table 1. In total, 2,861 men and women were included in the present study with highest participation from India (1,156) and lowest from South Africa (166). A greater proportion of the participants were female and in the age group of 50–69 years. In all countries, a strikingly low proportion of the participants reported having enough money to meet the needs completely. Overall, more than one-third of the participants reported their current health status as good (35.6%), and about a quarter as bad (26.3%). Approximately three-fifth expressed being satisfied with life (59.1%).

**Table 1:** Sample characteristics SAGE Wave-1.

	Pooled (*n* = 2,861)	China (*n* = 817)	Ghana (*n* = 412)	India (*n* = 1,156)	Russia (*n* = 310)	South Africa (*n* = 166)
Age groups						
50–59	25.6	7.6	30.6	38.2	30.0	57.8
60–69	42.4	88.1	29.1	34.3	24.5	25.9
70–79	15.8	3.9	26.2	20.9	32.3	13.9
≥79	16.2	0.4	14.1	6.5	13.2	2.4
Residency						
Urban	53.5	93.3	43.9	20.0	79.4	66.9
Rural	46.5	6.7	56.1	80.0	20.6	33.1
Sex						
Male	31.4	6.6	42.2	45.9	24.2	39.2
Female	68.6	93.4	57.8	54.1	75.8	60.8
Currently married						
Yes	38.6	4.3	58.0	30.3	58.7	54.2
No	61.4	95.7	42.0	69.7	41.3	45.8
Education						
Nil/less than primary school	33.0	4.8	59.5	63.1	1.3	22.9
Primary	16.4	5.4	21.4	22.8	13.5	53.0
Secondary	27.4	87.5	1.5	6.4	20.3	11.4
High school	23.2	2.3	17.7	7.8	64.8	12.7
Employed						
Yes	57.9	90.2	57.4	49.7	22.0	21.8
No	42.0	9.8	42.6	50.3	78.0	77.6
Ever used alcohol						
Yes	49.9	90.1	54.9	15.9	49.4	37.3
No	50.1	9.9	45.1	84.1	50.6	62.7
Income sufficiency						
Not at all	65.9	91.7	82.0	45.2	56.1	75.9
Mostly	31.2	7.5	17.5	50.0	37.1	23.5
Completely	2.9	0.9	0.5	4.8	6.8	0.6
Self-rated health						
Good	35.6	85.2	31.1	17.5	1.6	17.5
Moderate	38.1	5.6	49.3	50.1	42.6	48.8
Very bad	26.3	9.2	19.7	32.4	55.8	33.7
Satisfaction with life						
Satisfied	59.1	88.5	43.2	51.7	27.4	45.2
Neutral	25.6	6.7	34.2	33.8	39.4	25.9
Very dissatisfied	15.3	4.8	22.6	14.4	33.2	28.9

**Note:**

N.B. Figures denote percentages.

### Prevalence of engaging in physical activity

Table 2 shows the overall prevalence of taking >75 min of VPA/week (85.4%, (95% CI [81.3–88.7])), >150 min/week (61.6%, (95% CI [52.9–69.6])), occasional moderate leisure time physical activity (9.6%, (95% CI [7.2–12.6])) and walking/bike-riding (75.1%, (95% CI [68.7–80.6])). Prevalence of VPA was highest South Africa 97.1% (95% CI [93.0–98.8]) and that of MPA in China 86.4% (95% CI [51.4–97.4]). Taking LMPA as comparatively lower in all countries, with the highest being 47.1% (95% CI [38.5–56.0]) in Ghana and as low as 4.7% (95% CI [3.8–9.0]) in China. While the prevalence of walking/bike-riding was highest in China 91.2% (95% CI [62.7–98.5]) and lowest in South Africa 45.8% (95% CI [36.2–55.7]).

**Table 2:** Prevalence of taking four types PA. SAGE 2007–2010.

	Pooled	China	Ghana	India	Russia	South Africa
VPA						
≤75 min/week	14.6 [11.3–18.7]	13.6 [2.6–48.6]	3.4 [2.0–5.8]	18.9 [15.9–22.2]	7.6 [3.6–15.2]	2.9 [1.2–7.0]
≥75 min/week	85.4 [81.3–88.7]	86.4 [51.4–97.4]	96.6 [94.2–98.0]	81.1 [77.8–84.1]	92.4 [84.8–96.4]	97.1 [93.0–98.8]
MPA						
≤150 min/week	38.4 [30.4–47.1]	13.6 [2.6–48.6]	42.1 [35.1–49.4]	44.9 [40.5–49.5]	31.9 [22.1–43.6]	15.3 [9.8–23.1]
≥150 min/week	61.6 [52.9–69.6]	86.4 [51.4–97.4]	57.9 [50.6–64.9]	55.1 [50.5–59.5]	68.1 [56.2–77.9]	84.7 [76.3–90.2]
LMPA						
Yes	9.6 [7.2–12.6]	4.7 [3.8–9.0]	47.1 [38.5–56.0]	10.2 [8.1–12.8]	7.5 [3.2–16.7]	11.6 [6.1–20.8]
No	90.4 [87.4–92.8]	95.3 [75.6–99.2]	52.9 [43.9–61.3]	89.8 [87.2–91.9]	92.5 [83.3–96.8]	88.4 [79.2–93.9]
Walk/Bike						
Yes	75.1 [68.7–80.6]	91.2 [62.7–98.5]	75.0 [68.4–80.6]	73.6 [69.1–77.6]	69.5 [57.3–79.5]	45.8 [36.2–55.7]
No	24.9 [19.4–31.3]	8.8 [1.5–37.3]	25.0 [19.4–31.6]	26.4 [22.4–30.9]	30.5 [20.5–42.7]	54.2 [44.3–63.8]

**Note:**

N.B. Figures denote percentages. 95% CI were shown in square bracket.

Prevalence of ST and SA was, respectively, 31.0% (95% CI [24.3–38.7]) and 5.5% (95% CI [3.9–7.5]). Gender-stratified prevalence was presented in Table 3. Generally speaking, the proportion of participants with ST or SA was significantly lower than other four countries (ST: 5.4% in China vs. over 34% in each of the four countries; SA: 2.5% in China vs. 5.9% and over in each of the four countries). Within the country, it appeared that the prevalence of both ST (15.5% vs. 3.9%) and SA (11.1% vs. 1.2%) were significantly higher among men in China, while women had significantly higher rates of ST (25.7% vs. 41.7%) and SA (4% vs. & 7.6%) in India.

**Table 3:** Prevalence of suicidal thoughts and suicide attempts stratified by sex SAGE 2007–2010.

	Pooled	Men	Women	*p*-value
Suicidal thoughts	31.0 [24.3–38.7]	26.6 [21.8–31.9]	34.0 [23.1–46.8]	0.001
China	5.4 [1.1–22.1]	15.5 [8.4–27.0]	3.9 [0.6–19.9]	0.001
Ghana	43.2 [36.7–49.8]	43.4 [34.1–53.3]	43.0 [34.7–51.7]	0.460
India	34.1 [30.2–38.1]	25.7 [20.5–31.7]	41.7 [36.3–47.2]	0.000
Russia	39.8 [23.0–59.5]	37.8 [19.5–60.3]	40.4 [22.3–61.6]	0.170
South Africa	44.4 [35.3–53.9]	37.9 [27.9–49.0]	49.0 [36.5–61.6]	0.200
Suicide attempts	5.5 [3.9–7.5]	4.6 [3.1–6.9]	6.0 [3.8–9.4]	0.049
China	2.5 [0.5–11.2]	11.1 [5.1–22.8]	1.2 [0.2–7.0]	0.001
Ghana	8.1 [5.5–11.8]	9.1 [5.4–14.9]	7.3 [4.4–12.0]	0.220
India	5.9 [4.3–8.0]	4.0 [2.4–6.8]	7.6 [5.3–10.8]	0.020
Russia	4.2 [1.3–12.9]	1.8 [0.3–9.2]	4.9 [1.3–16.2]	0.190
South Africa	21.0 [15.1–28.4]	24.2 [16.0–34.7]	18.8 [11.3–29.5]	0.210

**Figure 1:**
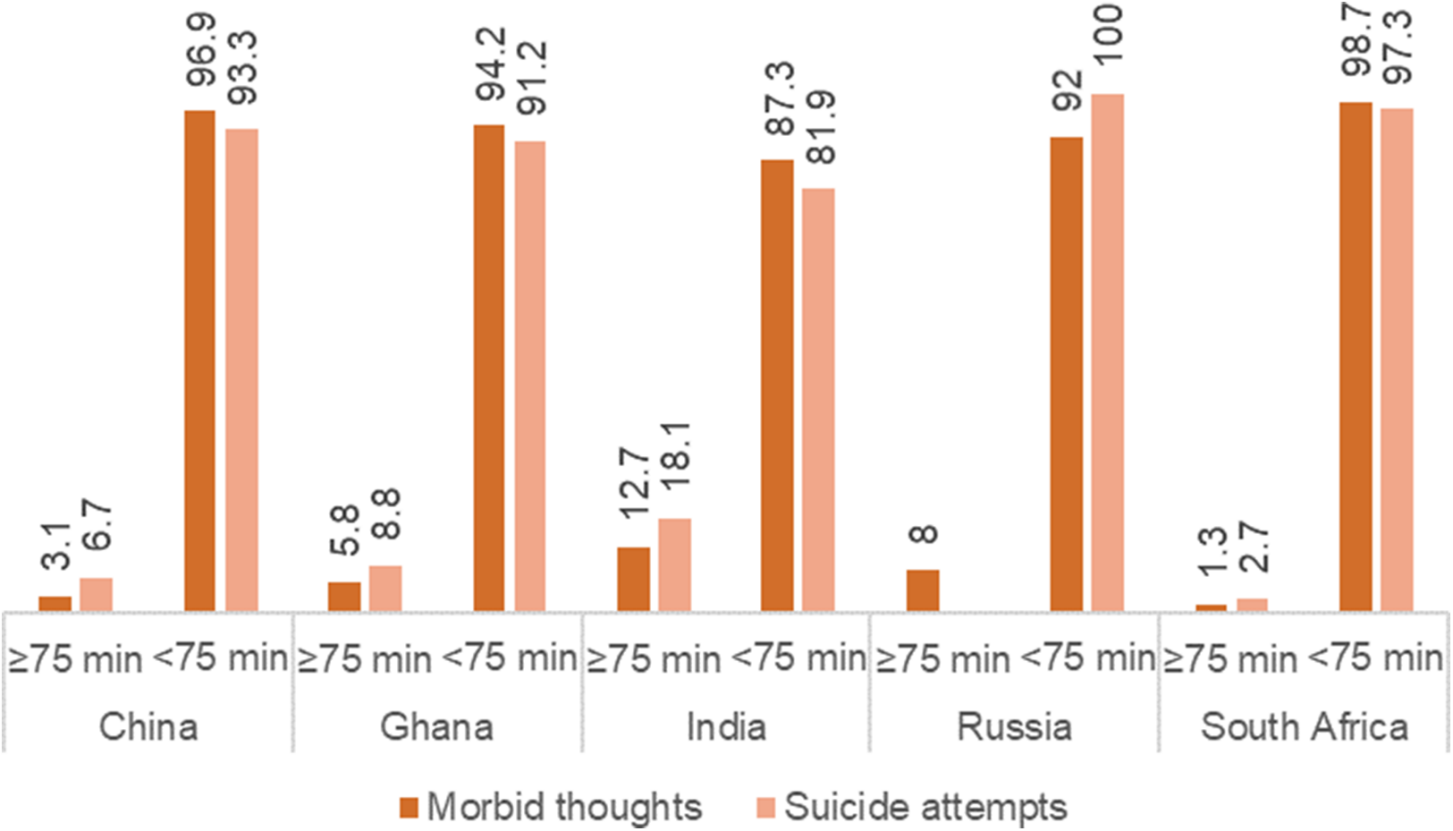
Prevalence of suicidal thoughts and suicide attempts stratified by patterns of VPA.

Figure 1 reveals a strong negative relationship between VPA and ST as well as SA (*p* < 0.05). The percentage of participants who reported having ST and SA was markedly higher among those did not take recommended levels of VPA.

**Figure 2:**
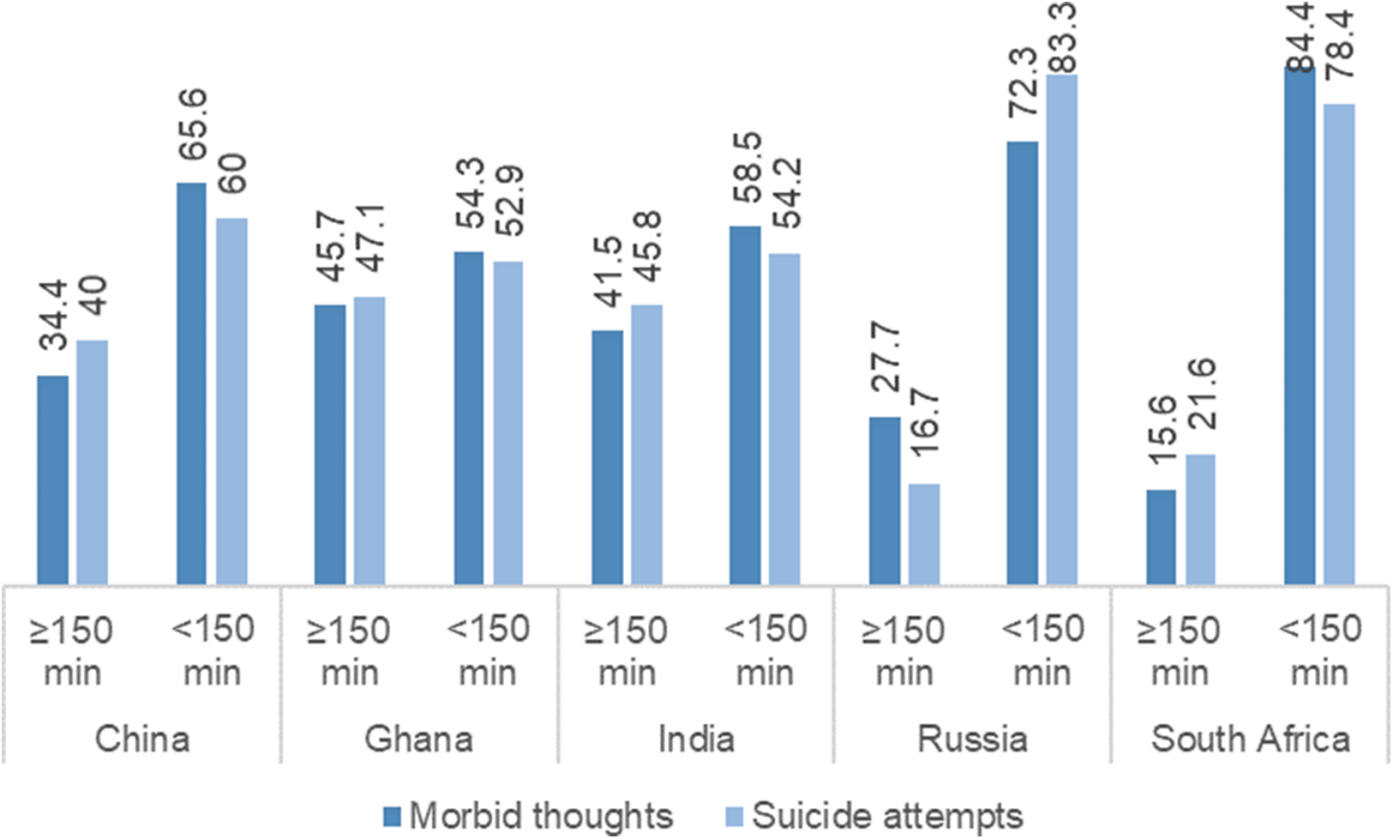
Prevalence of suicidal thoughts and suicide attempts stratified by patterns of MPA.

Figure 2 illustrates that the prevalence of both ST and SA was higher among participants who reported taking less than 150 min of MPA in all five countries, with the difference being more remarkable for Russia and South Africa.

**Figure 3:**
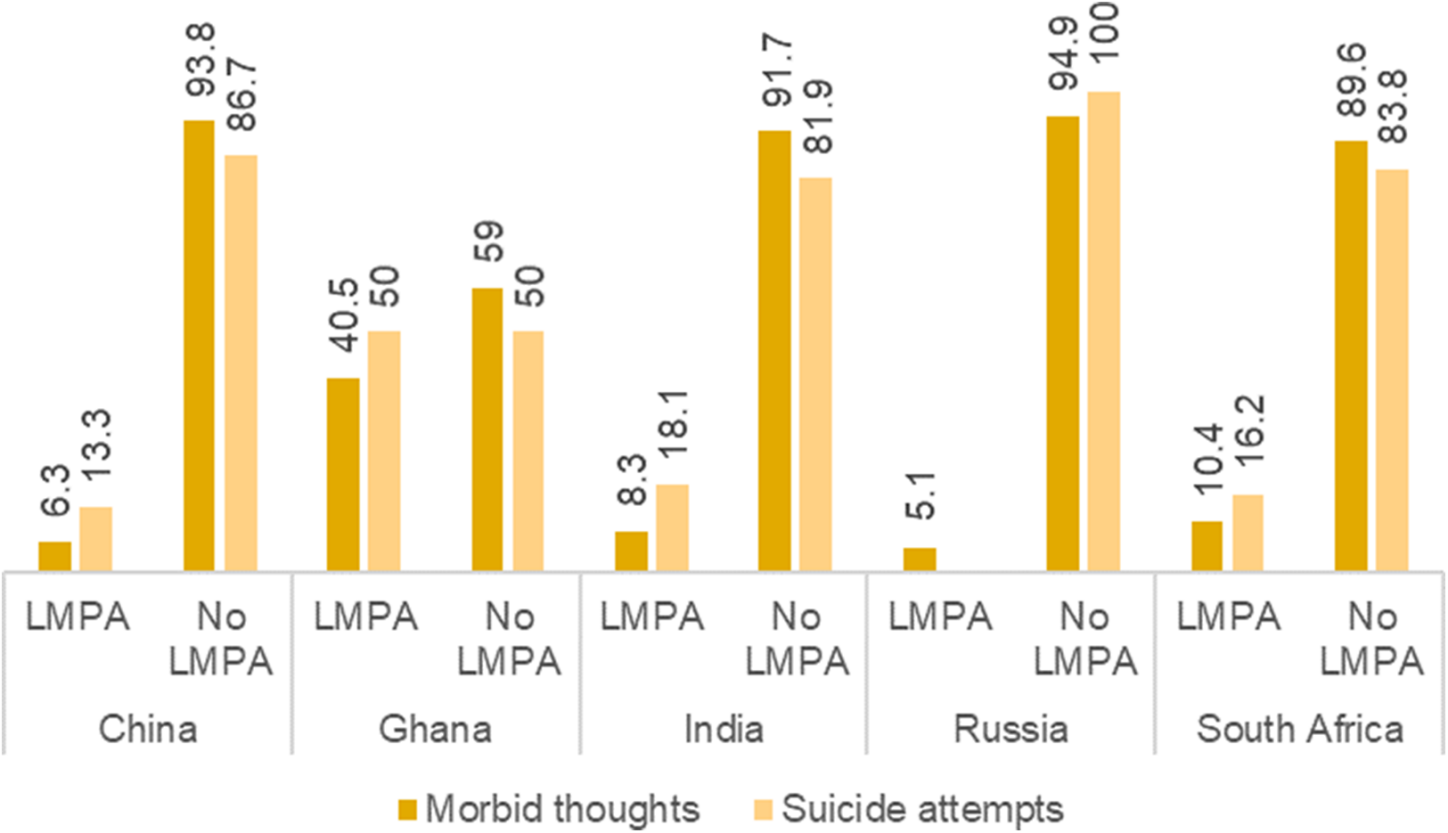
Prevalence of suicidal thoughts and suicide attempts stratified by patterns of LMPA.

Figure 3 indicates that participants who took LMPA had significantly lower prevalence of having ST and SA in all five countries. In Russia, all of the suicide ideation cases occurred among those who did not take any LMPA.

**Figure 4:**
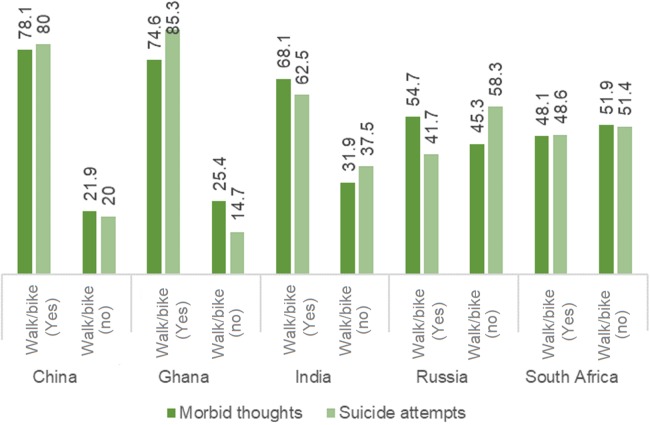
Prevalence of suicidal thoughts and suicide attempts stratified by patterns of walking/biking.

As per Figure 4, not walking/bike-riding had significantly positive association (*p* < 0.05) with ST and SA in all countries except in South Africa, with the association being most pronounced for China and Ghana.

### Multivariate association between self-reported ST/SA and PA

Findings from the multivariable regression analyses were summarized in Tables 4 and 5. From Tables 4 and 5 it is evident that in general, participants who did not engage in PA were more likely to report having ST and SA. In India, for instance, men and women who reported not being involved in VPA had 1.3 and 1.8 times higher odds of having ST and 1.4 and 2.6 times higher odds of having SA. MPA, LMPA and walking/bike-riding also appeared to be associated with both of the outcome variables, however, the associations varied in strength and across countries and gender. For instance, walking/bike-riding was significantly associated with having ST among both men in women only in Russia, and with SA only in China and South Africa. In Russia, not engaging in MPA (adjusted OR = 1.399) and LMPA (adjusted OR = 2.270) was associated with higher odds of experiencing ST among men, but not among women.

**Table 4:** Adjusted odds ratios of having suicidal thoughts for different type of PA. SAGE 2007–2010.

	Pooled		China		Ghana		India		Russia		South Africa	
	Male	Women	Men	Women	Men	Women	Men	Women	Men	Women	Men	Women
**VPA (>75 min/week)**												
≤75 min/week	**1.265**	**1.509**	0.947	**1.958**	0.885	**1.307**	**1.330**	**1.825**	**1.257**	1.028	**1.553**	**2.938**
**MPA (>150 min/week)**												
≤150 min/week	**1.292**	**1.449**	**1.670**	1.158	**1.444**	**1.327**	**2.251**	0.927	1.138	**1.399**	1.085	**1.315**
**LMPA (Yes)**												
No	1.012	**1.699**	0.885	**1.408**	**1.331**	**3.061**	0.952	1.228	1.674	**2.270**	**2.374**	**1.848**
**Walk/Bike (Yes)**												
No	1.125	**3.039**	0.219	0.718	0.818	0.947	1.197	1.137	**1.470**	**2.697**	**1.467**	1.085

**Note:**

N.B. ORs are adjusted for age, residency, sex, current marital status, education, employment, alcohol intake, self-rated health, self-rated life satisfaction; references categories are shown in parenthesis; bolded numbers are significant at *p* < 0.05.

**Table 5:** Adjusted odds ratios of having suicidal attempts for different type of PA. SAGE 2007–2010.

	Pooled	Pooled (sex-stratified)		China		Ghana		India		Russia		South Africa	
		Male	Women	Men	Women	Men	Women	Men	Women	Men	Women	Men	Women
**VPA (>75 min/week)**													
≤75 min/week	**1.265**	1.454	**1.526**	1.285	**1.770**	1.531	**2.889**	**1.433**	**2.555**	1.390	**1.697**	**1.155**	**1.940**
**MPA (>150 min/week)**													
≤150 min/week	**1.292**	1.366	**1.532**	1.021	**2.147**	**2.324**	0.761	**2.540**	**2.530**	1.092	**1.156**	**2.247**	**1.298**
**LMPA (Yes)**													
No	1.012	1.021	**1.475**	**2.429**	**1.770**	1.142	1.389	1.298	**1.430**	1.326	**2.045**	**1.433**	**1.871**
**Walk/Bike (Yes)**													
No	1.125	1.285	**1.392**	**3.047**	**1.595**	1.714	1.057	**1.944**	**2.021**	**1.944**	**1.838**	1.353	1.463

**Note:**

N.B. ORs are adjusted for age, sex, current marital status, education, employment, alcohol intake, self-rated health, self-rated life satisfaction; references categories are shown in parenthesis; bolded numbers are significant at *p* < 0.05.

## Discussion

### General discussion

On par with the high-income countries, suicide is becoming increasingly common phenomenon in the LMICs. Health and social researchers are responding to the rising burden of suicide rates through giving novel and perspectives and approaches to addressing the risk factors (Fowler, 2012; Milner et al., 2015). The validation of PA’s impact on LMICs may shed light on the identification of novel interventions with universal effectiveness besides the conventional restriction of access to lethal approaches and educating physicians in accurately diagnosis of depression (Mann et al., 2005). What is more, the findings provide a clearer vision regarding the policy design by reminding that PA interventions should be culture- and gender-specific. Some experience could be extracted from Chinese and Africa cultures: open air group activities such as Tai Chi and square dance are part of Chinese culture (Lu, Fu & Liu, 2016), whereas traditional dance is rooted in the culture of South Africa (Sedibe et al., 2014), and there appears to be peer support and influence on promoting PA in both types of exercise. Policy actions, such as India and China both created sports infrastructure at grassroots level and at the same time involved sectors/sports personnel to create a PA culture (Lachat et al., 2013), may provide some references for countries encountering similar problems.

### Prevalence of suicidal behaviors

The findings revealed high ST and SA prevalence among the elderly in LMICs, respectively, accounting for 31% and 5.5%. In particular, except those who lived in China, more than 43% of the elderly in Ghana, India, Russia and South Africa ever thought about suicide within 12 months before investigation. This figure is significantly higher than the corresponding findings from developed countries, where the ST rate ranges from 3% to 15.9% (Nock et al., 2008). Indeed, the rates for ST and SA in China were significantly lower compared to those in other four countries, the observations are in an agreement with prior studies against older adults in China (Li, Xu & Chi, 2016; Simon et al., 2013), where the rates of ST and SA were found to range from 1% to 16.7% and 0.35% to 7.6%, respectively. However, some researchers speculated that the ST/SA rate in China may be underreported due to Chinese culture: suicide is heavily stigmatized in traditional Chinese culture, which may result in elderly adults’ hesitation in reporting suicidal behaviors (Li, Xu & Chi, 2016; Ma et al., 2009). Indeed, there are some gaps between ST, SA and complete suicide, the strong association between these three elements together with the high prevalence of ST and SA observed in the present study still reminds the urgent need to raise attention to suicide risks among elderly people in LMICs.

Although the study did not tend to focus on gender difference related to suicidal ideation, some interesting findings were extracted. The prevalence of ST and SA were significantly higher among men in China, while it was the opposite scenario in India. The figure in China contradicts the findings from the studies carried out before 2010, which outlined a relatively low male-to-female ratio and indicated suicide a way for women in lower Socioeconomic status (SES) to protest unfair treatment that rooted in traditional Chinese culture (Yang et al., 2005). However, we noted that studies after 2010 tend to consider women a protective factor from suicidal ideation (Dong et al., 2014). This may be due to: (1) the dramatic development in the past decade that lays heavy social stress (in particular financial stress) on men, considering the cultural responsibility to support family (Wang, Chan & Yip, 2014; Zhong, Chiu & Conwell, 2016a); (2) the traditional gender role such as independence, and therefore, a weak intention to seek help for negative feelings such as loneliness (Zhong, Chiu & Conwell, 2016b). Regarding the male-to-female ratio in India, although contradicting the Western experience, it comforts to prior research findings in Indian context (Kar, 2010). Similar to Phillips & Hui (2012) statement, these interesting findings affirm that the risk factors of suicide vary greatly between cultures and over time.

### Physical activity

The findings revealed that the majority of participants met the WHO international guidelines regarding both VPA and MPA, and had experience with walk/bike-riding. This finding is consistent with evidence revealed by a systematic review, which indicates most studies reporting 20–60% of older adults with sufficient PA (Sun, Norman & While, 2013). However, the findings also outlined some significant variations across five countries. For instance, in China, above 90% of the participants reported walking/bike-riding for at least 10 min every day last week, while in South Africa less than half of the population did. The underlying causes behind these variations are not interpretable from the current findings. However, the possible explanations for high prevalence of walking/bike-riding in China might be the culture and governmental publicity: (1) bikes are still a common travel transportation for Chinese citizens in China especially in rural areas, although the past decade has witnessed a sharp increase in motorization (Feng, 2017); and (2) the Chinese government has launched “Ten Thousand Steps a Day” initiative for the Chinese elderly since 2007 (Zhang et al., 2016). The low prevalence of walking/bike-riding in South Africa might be due to the low neighborhood walkability: one study documented that only 54.2% of the participated middle and older-aged adults had sidewalks on most of the streets in their neighborhood, and only 47.8% had sidewalks in their neighborhood well maintained (Malambo et al., 2017).

### Association between physical activity and suicidal behaviors

Our findings revealed significantly negative relationships between all levels of PA and ST as well as SA, which is consistent with some prior studies (Cho, 2014; Davidson et al., 2013; Simon, Powell & Swann, 2004). In the bivariate analysis, percentage of participants who reported having ST and SA was found to be markedly higher among those did not take recommended levels of VPA and MPA, and these findings were similar for LMPA and walking/bike riding as well, although some variations across countries and among different types of PA were noted. For instance, ST and SA were more prevalent among the non-participants of LMPA in China, India, Russia and South Africa compared with that in Ghana. In a context where prevalence of LMPA was considerably low, the promoting LMPA is of great promise in these four countries. Similarly, VPA and MPA were found with stronger relationship with the prevalence of ST and SA in India and South Africa; Walk/bike-riding was found with stronger association with the prevalence of SA in China, India and Russia. Due to the lack of cross-national comparative studies on this issue, the underlying cause indeed awaits further investigation, however, some implications could be extracted from a study exploring the association between PA and mental health among adolescents in six other middle-income countries: this study also observed variations across countries and between different types of PA, and therefore, on the one hand, pointed out the difficulties in finding concrete underlying reasons; on the other hand, speculated that important information, such as social-network size, type (lone or group PA) and context of PA, not being included in the dataset may be the reason for the sample-specific variation (Arat & Wong, 2017). Therefore, the primary finding outlines the need for context-based interventions. For instance, combining the fact that over 50% of the respondents in South Africa did not took LPA, and these group of respondents had larger odds of ST than those who did, strategies such as encouraging regular PA and improving walkability are worth consideration.

Another important finding was the sex-difference in the association between different levels of PA and ST/SA was detected in women. This finding partly conforms with that derived from a study on Korean adults (aged 19 and older), where negative associations were observed between ST and PA above moderate intensity in male participants, however, an opposite result was observed in female participants (Song & Lee, 2016). Regarding the contradictory findings in female participants, the reason may be attributed to the age: prior studies speculated that women took PA for labor but exercise, whereas PA above moderate intensity may lead to ST/SA in women. This may be a different picture in our study: women aged 50 and above are less likely to carry out intensive labor activities, and therefore, they are more likely to take PA for exercise or social connection purposes. This may be supported by Heesch et al.’s (2016) study that indicated positive associations between PA and physical functioning, vitality and social functioning in mid-age and older women. This study further revealed that the associations were significantly stronger in older women compared to that in mid-age women. Moreover, given women’s more developed connectedness relative to elderly men (Fassberg et al., 2012), PA and the derived social connection may result in greater improvement in elderly women’s mental health, and therefore, reduce the risk of ST/SA to a larger extent. Similar findings can be retrieved from Salguero et al.’s (2011) study that indicated PA with a significantly higher value in improving elderly women’s mental health than that in improving elderly men’s mental health.

### Strengths and limitations

This study is an important contribution to the current literature. To the best of our knowledge, this study was the first one that examined ST and SA in relation to different levels of PA using a cross-national sample of the LMICs, and also one of the very few studies to estimate the ST and (or) SA against the elderly in LMICs. However, the findings need to be interpreted with caution. Surveys were conducted during 2007–2010 and may not represent the latest scenario in these countries. We gathered data for several countries to understand the pattern of the association across different sociocultural environments. Nonetheless, the sample were not representative of the individual countries, and hence the findings may not be generalizable for the entire population. The surveys were cross-sectional and hence the findings do not indicate causality or directionality of the associations. As the data were secondary, we had no control over the selection or measurement of the variables. We were also unable to estimate the precise number of hours/minutes for walking/bike-riding and LMPA due to data scarcity and had to categorize them as Yes/No rather than adequate/inadequate. Furthermore, the validity of the outcome measures (suicide ideation also remains to be known). Last but not least, data were self-reported, therefore remains subject to reporting bias and/or recall error.

## Conclusion

Based on analysis of secondary cross-sectional data from SAGE, the findings of this study conclude that PA plays an important role in the occurrence of ST and SA among elderly population. Not engaging in PA can significantly heighten the prevalence of suicidal behaviors among both men and women. Although the data were cross-sectional, and no linear dose-response relationship was observed, the findings provide important indications of potential harmful effects of no/inadequate PA on psychological morbidities among older individuals. Promoting adequate PA among older individuals through community-based programs can potentially contribute to reduction in the burden of PA in LMICs. Also, the combination of the prevalence of PA and the association between PA and suicidal behaviors may provide evidence for the target interventions for these countries. More studies are required to understand the culture- and gender-specific patterns of ST/SA and potentially viable interventions of individual countries by taking into consideration the unique contexts of each individual LMIC.

### Availability of data and materials

All data used in this study are available through the website of WHO SAGE program.

### Ethics approval and consent to participate

Demographic and Health Survey (DHS) surveys are approved by an independent review boards in the host country, and by ICF international. All participants gave prior informed consent to take part in the survey. Additional approval was not necessary as the data were anonymized and collected from public domain.

## Additional Information and Declarations

### Data Availability

The following information was supplied regarding data availability: The raw data came from the WHO Multi-Country Studies Data Archive (China, Ghana, India, Russia and South Africa): http://apps.who.int/healthinfo/systems/surveydata/index.php/catalog/sage.

## References

[ref-1] Ahmedani BK, Peterson EL, Hu Y, Rossom RC, Lynch F, Lu CY, Waitzfelder BE, Owen-Smith AA, Hubley S, Prabhakar D, Williams LK, Zeld N, Mutter E, Beck A, Tolsma D, Simon GE (2017). Major physical health conditions and risk of suicide. American Journal of Preventive Medicine.

[ref-2] Arat G, Wong PW-C (2017). The relationship between physical activity and mental health among adolescents in six middle-income countries: a cross-sectional study. Child & Youth Services.

[ref-3] Bailey M, McLaren S (2005). Physical activity alone and with others as predictors of sense of belonging and mental health in retirees. Aging & Mental Health.

[ref-4] Bishwajit G, O’Leary DP, Ghosh S, Yaya S, Shangfeng T, Feng Z (2017). Physical inactivity and self-reported depression among middle- and older-aged population in South Asia: world health survey. BMC Geriatrics.

[ref-5] Brown GK, Beck AT, Steer RA, Grisham JR (2000). Risk factors for suicide in psychiatric outpatients: a 20-year prospective study. Journal of Consulting and Clinical Psychology.

[ref-6] Brown DR, Blanton CJ (2002). Physical activity, sports participation, and suicidal behavior among college students. Medicine & Science in Sports & Exercise.

[ref-7] Carroll R, Metcalfe C, Gunnell D (2014). Hospital presenting self-harm and risk of fatal and non-fatal repetition: systematic review and meta-analysis. PLOS ONE.

[ref-8] Cho KO (2014). Physical activity and suicide attempt of South Korean adolescents—evidence from the eight Korea youth risk behaviors web-based survey. Journal of Sports Science and Medicine.

[ref-9] Conwell Y, Duberstein PR, Caine ED (2002). Risk factors for suicide in later life. Biological Psychiatry.

[ref-10] Davidson CL, Babson KA, Bonn-Miller MO, Souter T, Vannoy S (2013). The impact of exercise on suicide risk: examining pathways through depression, PTSD, and sleep in an inpatient sample of veterans. Suicide and Life-Threatening Behavior.

[ref-11] Deuter K, Procter N, Evans D, Jaworski K (2016). Suicide in older people: revisioning new approaches. International Journal of Mental Health Nursing.

[ref-12] Dong YH, Huang F, Hu GL, Liu Y, Zheng RZ, Zhang QH, Mao XQ (2014). The prevalence of suicidal ideation among the elderly in China: a meta-analysis of 11 cross-sectional studies. Comprehensive Psychiatry.

[ref-13] Du W-J, Tan J-P, Yi F, Zou Y-M, Gao Y, Zhao Y-M, Wang L-N (2015). Physical activity as a protective factor against depressive symptoms in older Chinese veterans in the community: result from a national cross-sectional study. Neuropsychiatric Disease and Treatment.

[ref-14] Elliot CA, Kennedy C, Morgan G, Anderson SK, Morris D (2012). Undergraduate physical activity and depressive symptoms: a national study. American Journal of Health Behavior.

[ref-15] Fassberg MM, Van Orden KA, Duberstein P, Erlangsen A, Lapierre S, Bodner E, Canetto SS, De Leo D, Szanto K, Waern M (2012). A systematic review of social factors and suicidal behavior in older adulthood. International Journal of Environmental Research and Public Health.

[ref-16] Feng JX (2017). The influence of built environment on travel behavior of the elderly in urban China. Transportation Research Part D-Transport and Environment.

[ref-17] Fowler JC (2012). Suicide risk assessment in clinical practice: pragmatic guidelines for imperfect assessments. Psychotherapy.

[ref-18] Hawton K, Heeringen KV (2009). Suicide. The Lancet.

[ref-19] Heesch KC, Van Gellecum YR, Burton NW, Van Uffelen JGZ, Brown WJ (2016). Physical activity and quality of life in older women with a history of depressive symptoms. Preventive Medicine.

[ref-20] Isacsson G, Rich CL, Jureidini J, Raven M (2010). The increased use of antidepressants has contributed to the worldwide reduction in suicide rates. British Journal of Psychiatry.

[ref-21] Kar N (2010). Profile of risk factors associated with suicide attempts: a study from Orissa, India. Indian Journal of Psychiatry.

[ref-22] Ku P-W, Fox KR, Chen L-J, Chou P (2012). Physical activity and depressive symptoms in older adults 11-Year Follow-Up. American Journal of Preventive Medicine.

[ref-23] Lachat C, Otchere S, Roberfroid D, Abdulai A, Maria F, Seret A, Milesevic J, Xuereb G, Candeias V, Kolsteren P (2013). Diet and physical activity for the prevention of noncommunicable diseases in low- and middle-income countries: a systematic policy review. PLOS Medicine.

[ref-24] Li H, Xu L, Chi I (2016). Factors related to Chinese older adults’ suicidal thoughts and attempts. Aging & Mental Health.

[ref-25] Lu J, Fu W, Liu Y (2016). Physical activity and cognitive function among older adults in China: a systematic review. Journal of Sport and Health Science.

[ref-26] Ma X, Xiang YT, Cai ZJ, Li SR, Xiang YQ, Guo HL, Hou YZ, Li ZB, Li ZJ, Tao YF, Dang WM, Wu XM, Deng J, Chan SSM, Ungvari GS, Chiu HFK (2009). Lifetime prevalence of suicidal ideation, suicide plans and attempts in rural and urban regions of Beijing, China. Australian & New Zealand Journal of Psychiatry.

[ref-27] Malambo P, Kengne AP, Lambert EV, De Villers A, Puoane T (2017). Association between perceived built environmental attributes and physical activity among adults in South Africa. BMC Public Health.

[ref-28] Mann JJ, Apter A, Bertolote J, Beautrais A, Currier D, Haas A, Hegerl U, Lonnqvist J, Malone K, Marusic A, Mehlum L, Patton G, Phillips M, Rutz W, Rihmer Z, Schmidtke A, Shaffer D, Silverman M, Takahashi Y, Varnik A, Wasserman D, Yip P, Hendin H (2005). Suicide prevention strategies: a systematic review. JAMA.

[ref-29] Milner AJ, Carter G, Pirkis J, Robinson J, Spittal MJ (2015). Letters, green cards, telephone calls and postcards: systematic and meta-analytic review of brief contact interventions for reducing self-harm, suicide attempts and suicide. British Journal of Psychiatry.

[ref-30] Nock MK, Borges G, Bromet EJ, Alonso J, Angermeyer M, Beautrais A, Bruffaerts R, Chiu WT, De Girolamo G, Gluzman S, De Graaf R, Gureje O, Haro JM, Huang Y, Karam E, Kessler RC, Lepine JP, Levinson D, Medina-Mora ME, Ono Y, Posada-Villa J, Williams D (2008). Cross-national prevalence and risk factors for suicidal ideation, plans and attempts. British Journal of Psychiatry.

[ref-31] Phillips MR, Hui GC (2012). The changing global face of suicide. The Lancet.

[ref-32] Runeson B, Tidemalm D, Dahlin M, Lichtenstein P, Langstrom N (2010). Method of attempted suicide as predictor of subsequent successful suicide: national long term cohort study. BMJ.

[ref-33] Salguero A, Martinez-Garcia R, Molinero O, Marquez S (2011). Physical activity, quality of life and symptoms of depression in community-dwelling and institutionalized older adults. Archives of Gerontology and Geriatrics.

[ref-34] Sedibe HM, Kahn K, Edin K, Gitau T, Ivarsson A, Norris SA (2014). Qualitative study exploring healthy eating practices and physical activity among adolescent girls in rural South Africa. BMC Pediatrics.

[ref-35] Shah A (2011). Elderly suicide rates: a replication of cross-national comparisons and association with sex and elderly age-bands using five year suicide data. Journal of Injury and Violence Research.

[ref-36] Simon M, Chang ES, Zeng P, Dong XQ (2013). Prevalence of suicidal ideation, attempts, and completed suicide rate in Chinese aging populations: a systematic review. Archives of Gerontology and Geriatrics.

[ref-37] Simon TR, Powell KE, Swann AC (2004). Involvement in physical activity and risk for nearly lethal suicide attempts. American Journal of Preventive Medicine.

[ref-38] Song H-B, Lee S-A (2016). Socioeconomic and lifestyle factors as risks for suicidal behavior among Korean adults. Journal of Affective Disorders.

[ref-39] Sturm J, Ploderl M, Fartacek C, Kralovec K, Neunhauserer D, Niederseer D, Hitzl W, Niebauer J, Schiepek G, Fartacek R (2012). Physical exercise through mountain hiking in high-risk suicide patients. A randomized crossover trial. Acta Psychiatrica Scandinavica.

[ref-40] Sun F, Norman IJ, While AE (2013). Physical activity in older people: a systematic review. BMC Public Health.

[ref-41] Thapa SB, Martinez P, Clausen T (2014). Depression and its correlates in South Africa and Ghana among people aged 50 and above: findings from the WHO Study on global ageing and adult health. Journal of psychiatry.

[ref-42] Turecki G, Brent DA (2016). Suicide and suicidal behaviour. The Lancet.

[ref-43] Unger JB (1997). Physical activity, participation in team sports, and risk of suicidal behavior in adolescents. American Journal of Health Promotion.

[ref-44] Vancampfort D, Hallgren M, Firth J, Rosenbaum S, Schuch FB, Mugisha J, Probst M, Van Damme T, Carvalho AF, Stubbs B (2018). Physical activity and suicidal ideation: a systematic review and meta-analysis. Journal of Affective Disorders.

[ref-45] Waern M, Rubenowitz E, Wilhelmson K (2003). Predictors of suicide in the old elderly. Gerontology.

[ref-46] Wang CW, Chan CLW, Yip PSF (2014). Suicide rates in China from 2002 to 2011: an update. Social Psychiatry and Psychiatric Epidemiology.

[ref-47] Wang HD, Naghavi M, Allen C, Barber RM, Bhutta ZA, Carter A, Casey DC, Charlson FJ, Chen AZ, Coates MM, Coggeshall M, Dandona L, Dicker DJ, Erskine HE, Ferrari AJ, Fitzmaurice C, Foreman K, Forouzanfar MH, Fraser MS, Pullman N, Gething PW, Goldberg EM, Graetz N, Haagsma JA, Hay SI, Huynh C, Johnson C, Kassebaum NJ, Kinfu Y, Kulikoff XR, Kutz M, Kyu HH, Larson HJ, Leung J, Liang XF, Lim SS, Lind M, Lozano R, Marquez N, Mensah GA, Mikesell J, Mokdad AH, Mooney MD, Nguyen G, Nsoesie E, Pigott DM, Pinho C, Roth GA, Salomon JA, Sandar L, Silpakit N, Sligar A, Sorensen RJD, Stanaway J, Steiner C, Teeple S, Thomas BA, Troeger C, VanderZanden A, Vollset SE, Wanga V, Whiteford HA, Wolock T, Zoeckler L, Abate KH, Abbafati C, Abbas KM, Abd-Allah F, Abera SF, Abreu DMX, Abu-Raddad LJ, Abyu GY, Achoki T, Adelekan AL, Ademi Z, Adou AK, Adsuar JC, Afanvi KA, Afshin A, Agardh EE, Agarwal A, Agrawal A, Kiadaliri AA, Ajala ON, Akanda AS, Akinyemi RO, Akinyemiju TF, Akseer N, Al Lami FH, Alabed S, Al-Aly Z, Alam K, Alam NKM, Alasfoor D, Aldhahri SF, Aldridge RW, Alegretti MA, Aleman AV, Alemu ZA, Alexander LT, Alhabib S, Ali R, Alkerwi A, Alla F, Allebeck P, Al-Raddadi R, Alsharif U, Altirkawi KA, Martin EA, Alvis-Guzman N, Amare AT, Amegah AK, Ameh EA, Amini H, Ammar W, Amrock SM, Andersen HH, Anderson B, Anderson GM, Antonio CAT, Aregay AF, Arnlov J, Arsenijevic VSA, Al A, Asayesh H, Asghar RJ, Atique S, Avokpaho E, Awasthi A, Azzopardi P, Bacha U, Badawi A, Bahit MC, Balakrishnan K, Banerjee A, Barac A, Barker-Collo SL, Barnighausen T, Barregard L, Barrero LH, Basu A, Basu S, Bayou YT, Bazargan-Hejazi S, Beardsley J, Bedi N, Beghi E, Belay HA, Bell B, Bell ML, Bello AK, Bennett DA, Bensenor IM, Berhane A, Bernabe E, Betsu BD, Beyene AS, Bhala N, Bhalla A, Biadgilign S, Bikbov B, Bin Abdulhak AA, Biroscak BJ, Biryukov S, Bjertness E, Blore JD, Blosser CD, Bohensky MA, Borschmann R, Bose D, Bourne RRA, Brainin M, Brayne CEG, Brazinova A, Breitborde NJK, Brenner H, Brewer JD, Brown A, Brown J, Brugha TS, Buckle GC, Butt ZA, Calabria B, Campos-Novato IR, Campuzano JC, Carapetis JR, Cardenas R, Carpenter D, Carrero JJ, Castaneda-Oquela CA, Rivas JC, Catala-Lopez F, Cavalleri F, Cercy K, Cerda J, Chen WQ, Chew A, Chiang PPC, Chibalabala M, Chibueze CE (2016). Global, regional, and national life expectancy, all-cause mortality, and cause-specific mortality for 249 causes of death, 1980–2015: a systematic analysis for the Global Burden of Disease Study 2015. The Lancet.

[ref-48] World Health Organization (2010). Global recommendations on physical activity for health.

[ref-49] World Health Organization (2014). Preventing suicide: A global imperative.

[ref-50] World Health Organization (2018). Suicide fact sheet. http://www.who.int/mediacentre/factsheets/fs398/en/.

[ref-51] Yang GH, Phillips MR, Zhou MG, Wang LJ, Zhang YP, Xu D (2005). Understanding the unique characteristics of suicide in China: national psychological autopsy study. Biomedical and Environmental Sciences.

[ref-52] Zhang Y, Li CY, Ding C, Zhao CL, Huang JZ (2016). The built environment and the frequency of cycling trips by Urban elderly: insights from Zhongshan, China. Journal of Asian Architecture and Building Engineering.

[ref-53] Zhong BL, Chiu HFK, Conwell Y (2016a). Elderly suicide trends in the context of transforming China, 1987–2014. Scientific Reports.

[ref-54] Zhong BL, Chiu HFK, Conwell Y (2016b). Rates and characteristics of elderly suicide in China, 2013–14. Journal of Affective Disorders.

